# Supplementary material for: Spike residue 403 affects binding of coronavirus spikes to human ACE2
Source: Nat Commun. 2021 Nov 25;12:6855. doi: 10.1038/s41467-021-27180-0 (PMC8617078; doi:10.1038/s41467-021-27180-0)
Supplement: Supplementary file 1 — Supplementary Information [file 41467_2021_27180_MOESM1_ESM.pdf]

Supplementary information for

Spike residue 403 affects binding of coronavirus spikes to human ACE2

Fabian Zech[1], Daniel Schniertshauer[1], Christoph Jung[2,3,4], Alexandra Herrmann[5], Arne Cordsmeier[5], Qinya Xie[1], Rayhane Nchioua[1], Caterina Prelli Bozzo[1], Meta Volcic[1], Lennart Koepke[1], Janis Müller[1], Jana Krüger[6], Sandra Heller[6], Steffen Stenger[7], Markus Hoffmann[8], Stefan Pöhlmann[8], Alexander Kleger[6], Timo Jacob[2,3,4], Karl-Klaus Conzelmann[8], Armin Ensser[5], Konstantin M.J. Sparrer[1] and Frank Kirchhoff[1*]

[1]Institute of Molecular Virology, Ulm University Medical Center, 89081 Ulm, Germany; [2]Institute of Electrochemistry, Ulm University, 89081 Ulm, Germany; [3]Helmholtz-Institute Ulm (HIU) Electrochemical Energy Storage, Helmholtz-Straße 16, 89081 Ulm, Germany; [4]Karlsruhe Institute of Technology (KIT), P.O. Box 3640, 76021 Karlsruhe, Germany; [5]Institute of Clinical and Molecular Virology, University Hospital Erlangen, Friedrich-Alexander Universität Erlangen-Nürnberg, 91054 Erlangen, Germany. [6]Department of Internal Medicine I, Ulm University Medical Center, 89081 Ulm, Germany; [7]Institute of Medical Microbiology and Hygiene, Ulm University Medical Centre, 89081 Ulm, Germany; [8]Infection Biology Unit, German Primate Center - Leibniz Institute for Primate Research, Göttingen, Germany; [9]Max von Pettenkofer-Institute of Virology, Medical Faculty, and Gene Center, Ludwig-Maximilians-Universität München, 81377 Munich, Germany.

* Address Correspondence to: Frank.Kirchhoff@uni-ulm.de

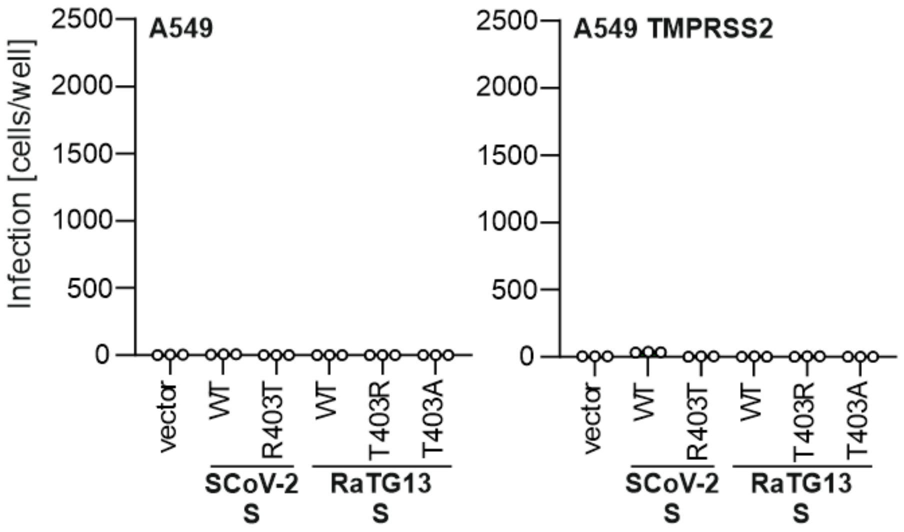

Supplementary Figure 1. A549 and A549 TMPRSS2 are not permissive for SCoV2 or RaTG13 S mediated infection. Automatic quantification of infection events of A549 and A549 TMPRSS2 expressing cells transduced with VSVΔG-GFP pseudotyped with SARS-CoV-2, RaTG13 or indicated mutant S. Bars represent the mean of three independent experiments (±SEM). Related to Fig. 2.

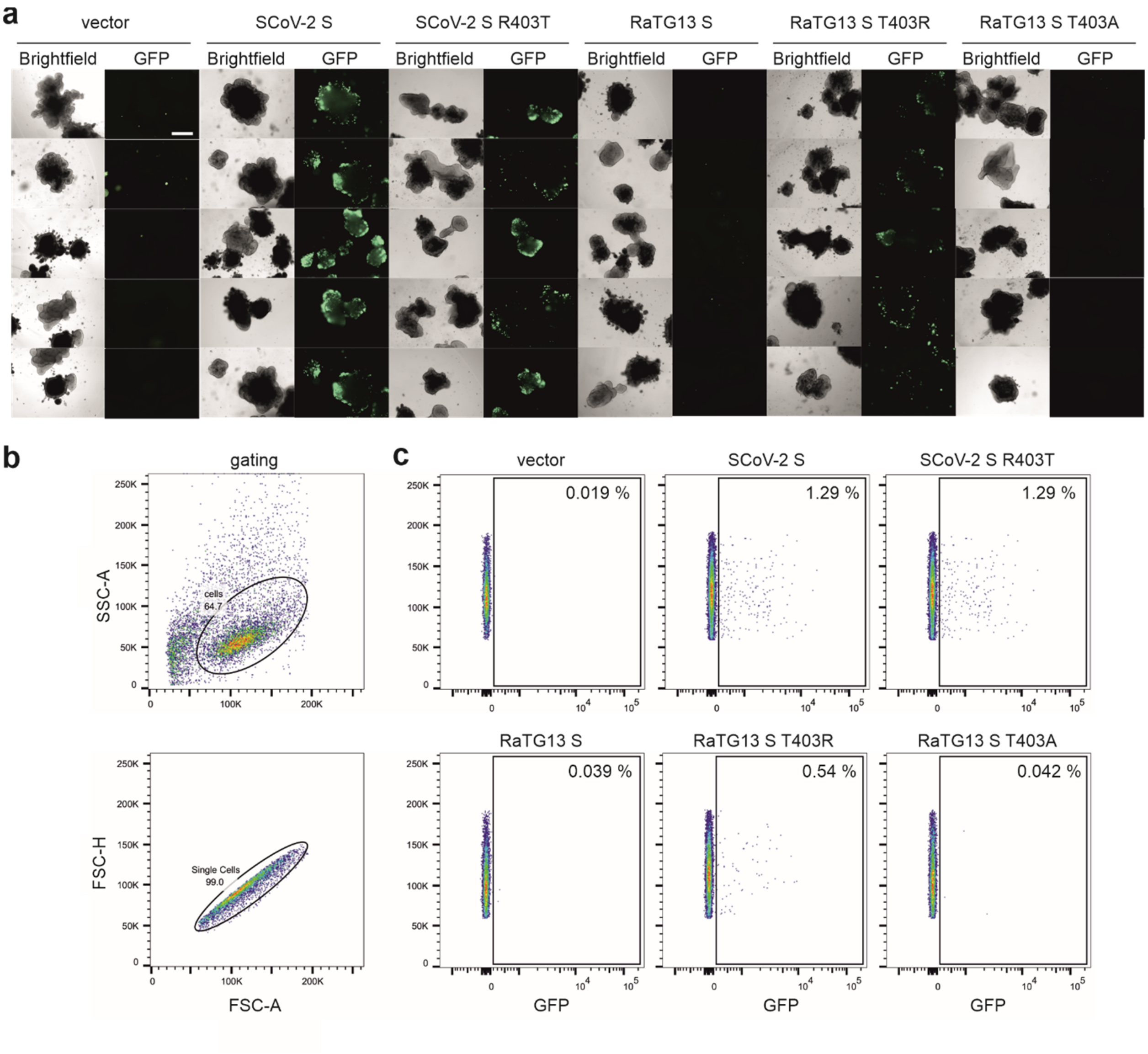

Supplementary Figure 2. T403R allows RaTG13 S to mediate infection of human intestinal organoids. a, Bright field and fluorescence microscopy (GFP) images of hPSC derived gut organoids infected with equal amounts of VSVΔG-GFP (green) pseudotyped with SARS-CoV-2, RaTG13 or indicated mutant S (2 h). Scale bar, 250µm. Pictures represent one of the independent experiments quantivied in Fig. 2d. **b,** Exemplary gating strategy of flow cytometry-based analysis of GFP-positive cells shown in panel a. Results of the quantitative analysis are provided in **Fig. 2d**.

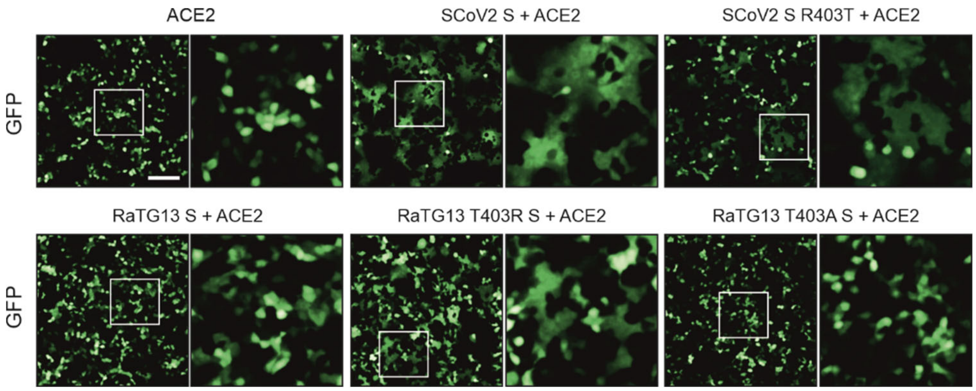

Supplementary Figure 3. T403R RaTG13 S allows ACE2 dependent cell fusion. Exemplary fluorescence microscopy images of HEK293T cells expressing SCoV2 S, RaTG13 S or the indicated mutant, Human ACE2 and GFP (green). Insets are indicated by white boxes. Scale bar, 125μm. The images represent a single experiment. Related to Fig. 2.

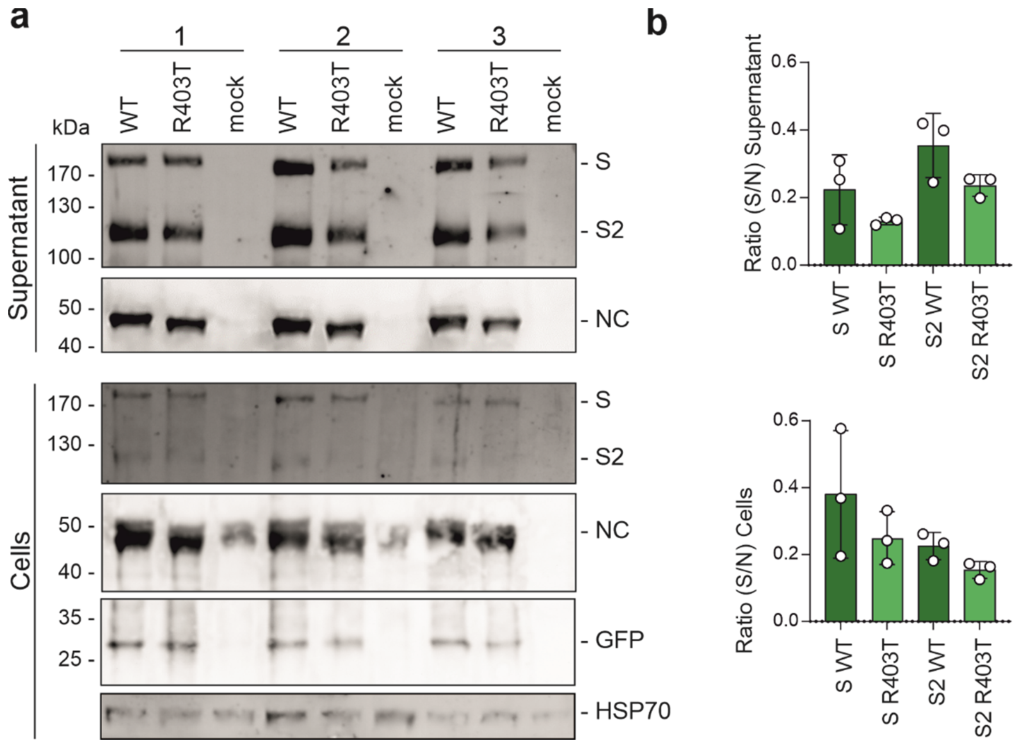

Supplementary Figure 4. Expression of WT and R403T S proteins by replication-competent SARS-CoV-2. a, Immunoblot of whole cells lysates (WCLs) and Supernatants of CaCo-2 cells infected with SARS-CoV-2 d6-YFP wild type or SARS-CoV-2 d6-YFP R403T. Blots were stained with anti-S, anti-N, anti-GFP and anti-HSP70 and quantified for Spike expression. n=3 (biological replicates). **b,** quantification of spike in the supernatant of SARS-CoV-2 d6-YFP wild type or SARS-CoV-2 d6-YFP R403T infected CaCo-2 cells. Bars represent the mean of three independent experiments (±SEM). Related to Fig. 2.

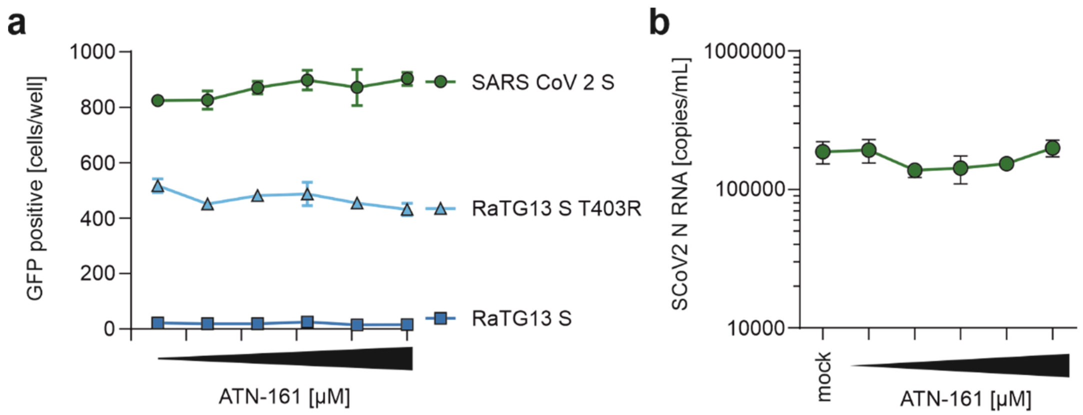

Supplementary Figure 5. SARS-CoV-2 entry is independent of α5β5 integrin. a, Automated quantification by GFP fluorescence of Caco-2 cells preincubated with indicated amounts of α5β5 integrin Inhibitor ATN-161 and infected with VSVΔG-GFP pseudotyped with SARS-CoV-2, RaTG13 T403R mutant or RaTG13 S. Lines represent the mean of three independent experiments (±SEM). **b,** quantification of viral RNA copies in the supernatant of Calu-3 cells preincubated with indicated amounts of ATN-161 and infected SARS-CoV-2 (MOI 0.05, 6 h). Lines represent the mean of three independent experiments (±SEM).

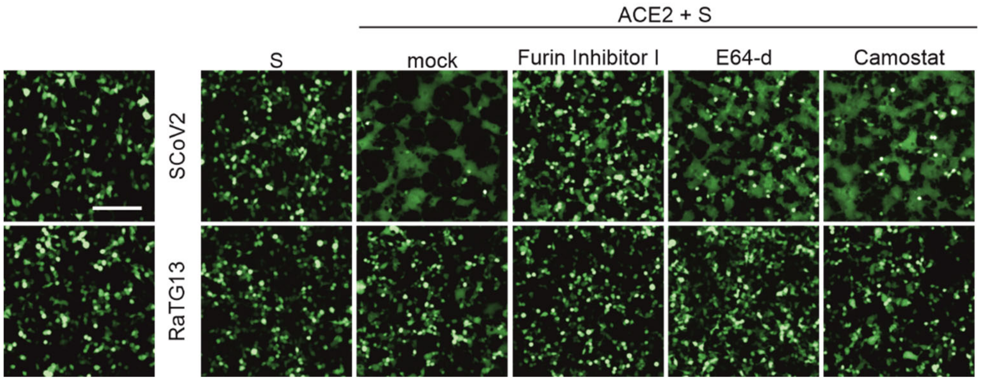

Supplementary Figure 6. Furin Inhibitor I and E64d but not Camostat inhibit SCoV-2 and RaTG13 S induced syncytia formation. Exemplary fluorescence microscopy images of HEK293T cells expressing SCoV2 S or RaTG13 S, Human ACE2 and GFP (green). The cells were incubated with the indicated protease inhibitor. The pictures represent a single experiment. Scale bar, 125µm. Related to Fig. 4.

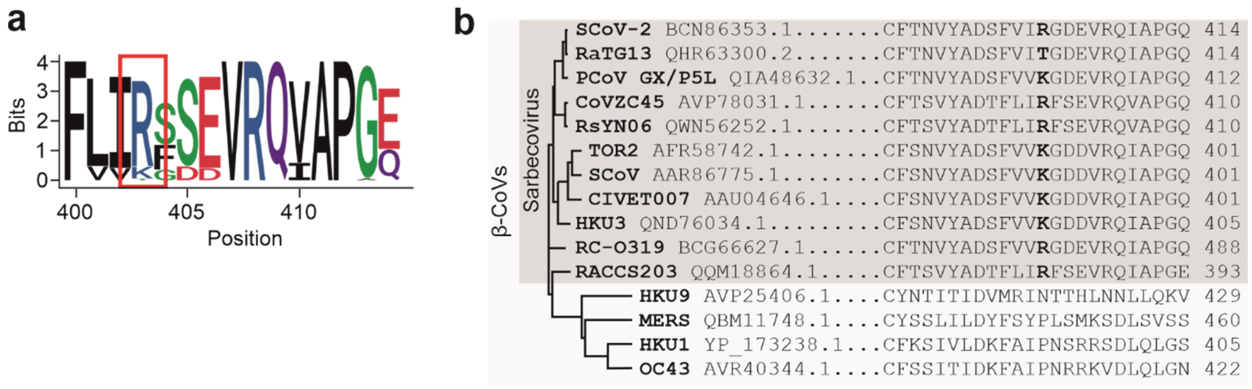

Supplementary Figure 7. Conservation of R403 in β-Coronavirus Spike proteins. a, Sequence logo of the alignment of 50 different Sarbecovirus Spike RBD sequences between sequence positions 400 and 414. Position R403 is highlighted by a red box. **b,** Primary sequence alignment of selected bat, pangolin and human coronaviruses. Position R403 is highlighted in bold.
