# Supplementary material for: Spike residue 403 affects binding of coronavirus spikes to human ACE2
Source: Nat Commun. 2021 Nov 25;12:6855. doi: 10.1038/s41467-021-27180-0 (PMC8617078; doi:10.1038/s41467-021-27180-0)
Supplement: Supplementary file 3 — Description of Additional Supplementary Files [file 41467_2021_27180_MOESM3_ESM.pdf]

**Description of Additional Supplementary Files**

File Name: Supplementary  Data 1
Description: **Statistical calculations and P-values**

File Name: Supplementary  Data 2
Description: **List of Primers**

File Name: Supplementary Movie 1
Description: Reactive molecular dynamic simulations of the interaction of SCoV-2 S R403 with Human ACE2 E37. Related to Fig. 1.

File Name: Supplementary Movie 2
Description: Reactive molecular dynamic simulations of the interaction of SCoV-2 S R403T mutant T403 with Human ACE2 E37. Related to Fig. 1.

File Name: Supplementary Movie 3
Description: Reactive molecular dynamic simulations of the interaction of RatG13 S T403 with Human ACE2 E37. Related to Fig. 1.

File Name: Supplementary Movie 4
Description: Reactive molecular dynamic simulations of the interaction of RatG13 T403R mutant R403 with Human ACE2 E37. Related to Fig. 1.
