# Supplementary material for: Spike residue 403 affects binding of coronavirus spikes to human ACE2
Source: Nat Commun. 2021 Nov 25;12:6855. doi: 10.1038/s41467-021-27180-0 (PMC8617078; doi:10.1038/s41467-021-27180-0)
Supplement: Supplementary file 10 — Reporting Summary [file 41467_2021_27180_MOESM10_ESM.pdf]

Corresponding author(s): Frank Kirchhoff

Last updated by author(s): 01.11.2021

Reporting Summary

Nature Research wishes to improve the reproducibility of the work that we publish. This form provides structure for consistency and transparency in reporting. For further information on Nature Research policies, see our Editorial Policies and the Editorial Policy Checklist.

Statistics

For all statistical analyses, confirm that the following items are present in the figure legend, table legend, main text, or Methods section.

n/a	Confirmed	
☐	☒	The exact sample size (*n*) for each experimental group/condition, given as a discrete number and unit of measurement
☐	☒	A statement on whether measurements were taken from distinct samples or whether the same sample was measured repeatedly
☐	☒	The statistical test(s) used AND whether they are one- or two-sided<br>*Only common tests should be described solely by name; describe more complex techniques in the Methods section.*
☒	☐	A description of all covariates tested
☒	☐	A description of any assumptions or corrections, such as tests of normality and adjustment for multiple comparisons
☐	☒	A full description of the statistical parameters including central tendency (e.g. means) or other basic estimates (e.g. regression coefficient) AND variation (e.g. standard deviation) or associated estimates of uncertainty (e.g. confidence intervals)
☐	☒	For null hypothesis testing, the test statistic (e.g. *F*, *t*, *r*) with confidence intervals, effect sizes, degrees of freedom and *P* value noted<br>*Give P values as exact values whenever suitable.*
☒	☐	For Bayesian analysis, information on the choice of priors and Markov chain Monte Carlo settings
☒	☐	For hierarchical and complex designs, identification of the appropriate level for tests and full reporting of outcomes
☒	☐	Estimates of effect sizes (e.g. Cohen's *d*, Pearson's *r*), indicating how they were calculated

Our web collection on statistics for biologists contains articles on many of the points above.

Software and code

Policy information about availability of computer code

Data collection	BD FACSDiva™ Version 8.0, Odyssey LI-COR Image Studio Lite Version 5.2, Cytation 3
Data analysis	BioTek Gen5, GraphPad Prism Version 9.2.0, Corel DRAW 2021,Odyssey LI-COR Image Studio Lite Version 5.2, Fiji 1.53, FlowJo 10, ClustalW in msa (1.18.0) , ggplot2 (3.3.5), ggseqlogo (0.1), Amsterdam Modeling Suite 2020, VMD 1.9.3, R (3.6.1)

For manuscripts utilizing custom algorithms or software that are central to the research but not yet described in published literature, software must be made available to editors and reviewers. We strongly encourage code deposition in a community repository (e.g. GitHub). See the Nature Research guidelines for submitting code & software for further information.

Data

Policy information about availability of data

All manuscripts must include a data availability statement. This statement should provide the following information, where applicable:

- Accession codes, unique identifiers, or web links for publicly available datasets
- A list of figures that have associated raw data
- A description of any restrictions on data availability

The datasets generated during and/or analyzed during the current study are available from the corresponding authors on request. All Source data are provided in the Supplementary Information/Source Data file. The SARS-CoV-2 S structure used in this study is available in the Protein Data Bank (PDB) under accession code 7KNB (https://www.rcsb.org/structure/7KNB); Primary coronavirus sequences are available from the National Center for Biotechnology and Information (NCBI) : https://www.ncbi.nlm.nih.gov/nuccore/(GU190215.1/QPD89842.1/KY417145.1/KY938558.1/KU182964.1/KJ473811.1/MN996532.1/KY417152.1/MK211376.1/KY417146.1/KY417150.1/KT444582.1/MG772933.1/MG772934.1/KY770858.1/KY770859.1/KJ473816.1/MK211374.1/JX993987.1/KJ473814.1/DQ648856.1/KY770860.1/KJ473812.1/KJ473813.1/JX993988.1/KY417143.1/MK211378.1/DQ412043.1/DQ648857.1/KY417148.1/MK211375.1/KY417147.1/KY417142.1/

MK211377.1/KJ473815.1/GQ153542.1/GQ153543.1/KF294457.1/GQ153547.1/DQ084199.1/GQ153548.1/DQ022305.1/DQ084200.1/GQ153545.1/GQ153546.1/GQ153539.1/GQ153540.1/GQ153541.1/GQ153544.1)

Field-specific reporting

Please select the one below that is the best fit for your research. If you are not sure, read the appropriate sections before making your selection.

☒ Life sciences ☐ Behavioural & social sciences ☐ Ecological, evolutionary & environmental sciences

For a reference copy of the document with all sections, see nature.com/documents/nr-reporting-summary-flat.pdf

Life sciences study design

All studies must disclose on these points even when the disclosure is negative.

Sample size	No sample size calculations were performed. Based on previous experiences, results were confirmed in at least three independent experiments.
Data exclusions	no data was excluded
Replication	Experiment were usually performed at least three times. Number of successful independent repeats are indicated in the figure legends.
Randomization	Randomization was not applicable for this study, no in vivo data was analysed or clinical trials performed. Selection or Subject bias/allocation bias not applicable.
Blinding	Blinding was not applicable as no manual readouts were performed. Human sera were used blinded.

Reporting for specific materials, systems and methods

We require information from authors about some types of materials, experimental systems and methods used in many studies. Here, indicate whether each material, system or method listed is relevant to your study. If you are not sure if a list item applies to your research, read the appropriate section before selecting a response.

Materials & experimental systems

n/a	Involved in the study	
☐	☒	Antibodies
☐	☒	Eukaryotic cell lines
☒	☐	Palaeontology and archaeology
☒	☐	Animals and other organisms
☐	☒	Human research participants
☒	☐	Clinical data
☒	☐	Dual use research of concern

Methods

n/a	Involved in the study	
☒	☐	ChIP-seq
☐	☒	Flow cytometry
☒	☐	MRI-based neuroimaging

Antibodies

Antibodies used

α-SARS-CoV-2 N Sino Biologicals #40588-V08B (1:1000)
α-SARS-CoV-2 S (1A9) GeneTex #GTX632604 (1:1000)
α-ACE2 Abcam, #ab108252 (1:1,000)
α-V5 rb Cell Signaling #13202 (1:1000)
α-V5 ms Cell Signaling, #80076 (1:1000)
VSV-M (23H12) Absolute Antibody, #Ab01404-2.0 (1:2000)
anti-nucleocapsid Invitrogen, #ARC2372 (1:2000)
Anti-HSP70 Santa Cruz W27/sc-24 (1:1000)
Anti-GFP GenScript A01704-40 (1:1000)
α-GAPDH Biolegend #631401 (1:5000)
MS-HRP RayBio (1:1,000)
Anti-human-488 Invitrogen A11013 (1:2000)
Anti-mouse-647 Invitrogen A31571 (1:2000)
Anti-rabbit-647 Invitrogen A21206 (1:2000)

IRDye® 800CW Goat anti-Mouse IgG Secondary Antibody, Li-CORE, Cat#926-32210, lot C20808-02, dilution (1:10000)
IRDye® 800CW Goat anti-Rat IgG Secondary Antibody, Li-CORE, Cat#926-32219, C91211-09, dilution (1:10000)
IRDye® 680CW Goat anti-Rabbit IgG Secondary Antibody,Li-CORE, Cat#925-68071, lot C806605-11, dilution (1:10000)
IRDye® 680CW Goat anti-Mouse IgG Secondary Antibody,Li-CORE, Cat#926-68070, lot C90910-21, dilution (1:10000)

	IRDye® 800CW Goat anti-Rabbit IgG Secondary Antibody,Li-CORE, Cat#926-32211, lot C70926-05, dilution (1:10000)
Validation	Antibodies were validated by the manufacturer as stated on the respective websites of the manufacturers.<br>https://www.sinobiological.com/recombinant-proteins/2019-ncov-cov-nucleocapsid-40588-v08b<br>https://www.genetex.com/Product/Detail/SARS-CoV-SARS-CoV-2-COVID-19-spike-antibody-1A9/GTX632604<br>https://www.abcam.com/ace2-antibody-epr44352-ab108252.html<br>https://www.cellsignal.com/products/primary-antibodies/v5-tag-d3h8q-rabbit-mab/13202<br>https://www.cellsignal.com/products/primary-antibodies/v5-tag-e9h8o-mouse-mab/80076<br>https://absoluteantibody.com/product/anti-vsv-m-23h12/<br>https://www.thermofisher.com/antibody/product/SARS-CoV-2-Nucleocapsid-Antibody-clone-ARC2372-Recombinant-Monoclonal/MA5-36086<br>https://www.scbt.com/de/p/hsp-70-hsc-70-antibody-w27<br>https://www.genscript.com/gsfiles/catalog/GFP_Antibody_flyer_04112011.pdf<br>https://www.antibodypedia.com/gene/3923/GAPDH/antibody/533961/631401<br>https://www.raybiotech.com/covid-19-spike-ace2-binding-assay-kit-en/<br>https://www.thermofisher.com/antibody/product/Goat-anti-Human-IgG-H-L-Cross-Adsorbed-Secondary-Antibody-Polyclonal/A-11013<br>https://www.thermofisher.com/antibody/product/Donkey-anti-Mouse-IgG-H-L-Highly-Cross-Adsorbed-Secondary-Antibody-Polyclonal/A-31571<br>https://www.thermofisher.com/antibody/product/Donkey-anti-Rabbit-IgG-H-L-Highly-Cross-Adsorbed-Secondary-Antibody-Polyclonal/A-21206<br>https://www.licor.com/bio/reagents/irdye-800cw-goat-anti-mouse-igg-secondary-antibody<br>https://www.licor.com/bio/reagents/irdye-800cw-goat-anti-rat-igg-secondary-antibody<br>https://www.licor.com/bio/reagents/irdye-680rd-goat-anti-rabbit-igg-secondary-antibody<br>https://www.licor.com/bio/reagents/irdye-680rd-goat-anti-mouse-igg-secondary-antibody<br>https://www.licor.com/bio/reagents/irdye-800cw-goat-anti-rabbit-igg-secondary-antibody

Eukaryotic cell lines

Policy information about cell lines

Cell line source(s)	Human HEK293T cells ATCC Cat# CRL-3216<br>Human epithelial colorectal adenocarcinoma cells (Caco 2)cells ATCC<br>Human epithelial lung adenocarcinoma cells (Calu-3 cells) ATCC<br>Human adenocarcinomic alveolar basal epithelial cells A549, A549 ACE2, A549 TMPRSS2 and A549 ACE2/TMPRSS2 provided and validated by Prof. Pöhlmann, German Primate Center, Göttingen. Original source A549: ATCC.<br>HUES8 (Derived from human blastocytes and provided by Harvard University, Cambridge, MA, provided by Alexander Kleger, Ulm University Medical Center)<br>Mouse I1 Hybridoma CRL-2700; ATCC<br>Tadarida brasiliensis derived lung epithelial (Tb 1 Lu) were provided, established and validated by Marcel A. Müller, Charité-Institute of Virology, Berlin<br>Rhinolophus affinis derived lung epithelial cells expressing human ACE2 (Ri 1 Lu huACE2) were provided, established and validated by Marcel A. Müller Charité-Institute of Virology, Berlin
Authentication	The cell lines were authenticated by ATCC, NIH or their lab of origin and not validated further in our laboratory. Specifications of authentification are available from the manufacturer or laboratories of origin.
Mycoplasma contamination	Cells were tested routinely to be free of mycoplasma using a PCR based test.
Commonly misidentified lines<br>(See ICLAC register)	no commonly misidentified cell lines were used in this study.

Human research participants

Policy information about studies involving human research participants

Population characteristics	ChAdOx1-nCoV-19/BNT162b2 vaccinated individuals were between 25 and 60 years old (average age: 34.5) with 58 % female participation. 2x BNT162b2 vaccinated individuals were between 22 and 60 years old (average age: 39.9) with 44 % female participation.
Recruitment	After vaccination, emplyees of ulm university medical center who expressed interest in participating in the current study were contacted. This recruiting biasses the cohort to mostly young adults and further self-selection bias can not be excluded. However, this should not affect the results of the current study.
Ethics oversight	For human stem cells used to generate gut organoids: Robert-Koch Institute: Approval according to the stem cell law 29.04.2020/AZ 3.04.02/0084. a more detailed description can be found in the Materials & Methods section of the manuscript. Blood samples of ChAdOx1-nCoV-19/BNT162b2 and BNT162b2 vaccinated individuals were obtained after the participant's information and written consent. Ethics approval was given by the Ethic Committee of Ulm University (vote 99/21 – FSt/Sta).

Note that full information on the approval of the study protocol must also be provided in the manuscript.

Flow Cytometry

Plots

Confirm that:

☒ The axis labels state the marker and fluorochrome used (e.g. CD4-FITC).

☒ The axis scales are clearly visible. Include numbers along axes only for bottom left plot of group (a 'group' is an analysis of identical markers).

☒ All plots are contour plots with outliers or pseudocolor plots.

☒ A numerical value for number of cells or percentage (with statistics) is provided.

Methodology

Sample preparation	For FACS preparation, the matrigel was dissolved and the extracted organoids were dissolved in Accutase (Stemcell technologies). The cells were fixed with PFA 1 % for 10 min at 4°C and washed with cold PBS containing 2% FBS. Flow cytometry analyses were performed using a FACS CANTO II (BD) flow cytometer. Transduction rates were determined by GFP expression
Instrument	FACS CantoII; BD
Software	FlowJow 10, LLC
Cell population abundance	Single living cells (90%)
Gating strategy	The gating strategy is provided in Supplementary Figure 2.

☒ Tick this box to confirm that a figure exemplifying the gating strategy is provided in the Supplementary Information.
